# Quantification of HER1, HER2 and HER3 by time-resolved Förster resonance energy transfer in FFPE triple-negative breast cancer samples

**DOI:** 10.1038/s41416-019-0670-8

**Published:** 2019-12-03

**Authors:** Alexandre Ho-Pun-Cheung, Hervé Bazin, Florence Boissière-Michot, Caroline Mollevi, Joëlle Simony-Lafontaine, Emeline Landas, Jean-Pierre Bleuse, Thierry Chardès, Jean-François Prost, André Pèlegrin, William Jacot, Gérard Mathis, Evelyne Lopez-Crapez

**Affiliations:** 1 ICM, Institut régional du Cancer de Montpellier, Montpellier, France; 2 Institut de Recherche en Cancérologie de Montpellier (IRCM), INSERM, Université de Montpellier, Institut régional du Cancer de Montpellier (ICM), Montpellier, France; 3 CisBio SA, Codolet, France; 4 GamaMabs Pharma SA, Centre Pierre Potier, Toulouse, France

**Keywords:** Breast cancer, Prognostic markers

## Abstract

**Background:**

Triple-negative breast cancer (TNBC) has a worse prognosis compared with other breast cancer subtypes, and biomarkers to identify patients at high risk of recurrence are needed. Here, we investigated the expression of human epidermal receptor (HER) family members in TNBC and evaluated their potential as biomarkers of recurrence.

**Methods:**

We developed Time Resolved-Förster Resonance Energy Transfer (TR-FRET) assays to quantify HER1, HER2 and HER3 in formalin-fixed paraffin-embedded (FFPE) tumour tissues. After assessing the performance and precision of our assays, we quantified HER protein expression in 51 TNBC specimens, and investigated the association of their expression with relapse-free survival.

**Results:**

The assays were quantitative, accurate, and robust. In TNBC specimens, HER1 levels ranged from ≈4000 to more than 2 million receptors per cell, whereas HER2 levels varied from ≈1000 to 60,000 receptors per cell. HER3 expression was very low (less than 5500 receptors per cell in all samples). Moderate HER2 expression was significantly associated with higher risk of recurrence (HR = 3.93; *P* = 0.003).

**Conclusions:**

Our TR-FRET assays accurately quantify HER1, HER2 and HER3 in FFPE breast tumour specimens. Moderate HER2 expression may represent a novel prognostic marker in patients with TNBC.

## Background

The term triple-negative breast cancers (TNBC) was first used in 2005^1^ to describe a subset of tumours characterised by absence or low levels of expression of oestrogen receptor (ER), progesterone receptor (PgR), and human epidermal growth factor receptor 2 (HER2). The lack of ER, PgR and HER2 overexpression rules out the use of hormonal therapies or anti-HER2 agents in TNBC. For this reason, systemic treatment of TNBC was limited to chemotherapy till the recent introduction of poly-ADP ribose polymerase (PARP) inhibitors for BRCA-deficient tumours.^2^ Recurrence is more frequent and the 5-year survival rate is lower in patients with TNBC than other breast cancer subtypes (34% versus 20%, and 77% versus 93%, respectively).^3,4^ Therefore, due to their poor prognosis and scarcity of targeted therapies, actionable molecular targets need to be identified.

Histologically, most TNBC share common characteristics, and 95% of them are classified as invasive ductal carcinomas.^5^ At the molecular level, Lehmann et al.,^6^ and more recently, Burstein et al.^7^ have described molecular subtypes of TNBC with distinct outcomes and drug sensitivities.^6–8^ While additional studies are needed to determine whether the different TNBC subtypes can be targeted with specific therapies, these “omics” analyses demonstrated that TNBC should not be considered as a single clinical entity that can be uniformly treated.

Given TNBC molecular heterogeneity, targeting tumour-specific alterations could significantly improve the outcome of the 60–70% of patients with TNBC who do not fully respond to chemotherapy.^5^ As dysregulated expression of human epidermal receptor (HER) family members is frequent in breast cancer, and given their crucial role in proliferation,^9^ these receptors have been extensively investigated as targets for anticancer therapy, particularly HER1, HER2 and HER3. HER1 overexpression is frequent in TNBC and is associated with poor clinical outcome.^10^ Preclinical studies demonstrated the sensitivity of TNBC cell lines to HER1 inhibitors, providing the rationale to test the efficacy of HER1-targeting agents in patients with TNBC.^11^ However, clinical studies remain inconclusive, possibly due to the lack of patient selection, because anti-HER1 targeted therapies were not restricted to patients with HER1-overexpressing tumours or patients without KRAS/NRAS mutations.^12^ Some recent studies suggested that patients with TNBC, which is, by definition, characterised by absence of HER2 overexpression, could benefit from anti-HER2 therapies, such as trastuzumab.^13,14^ This issue was specifically addressed by the National Surgical Adjuvant Breast and Bowel Project B-47 (NSABP B-47) trial that was designed to evaluate trastuzumab effect in 3270 women with breast cancers with low HER2 levels (IHC 1+ or 2+ and/or negative by FISH).^15^ This randomised trial did not find any improvement in invasive disease-free survival or overall survival in patients treated with trastuzumab and standard chemotherapy versus chemotherapy alone.^16^ Nevertheless, a recent study reported that in patients with TNBC, moderate HER2 expression (IHC score of 2+) correlates with relapse,^17^ suggesting that the clinical significance of different degrees of HER2 expression should be thoroughly explored in TNBC. Finally, the therapeutic potential of anti-HER3 agents for cancer treatment has been less investigated because HER3 kinase domain is defective. However, HER3 expression could be a prognostic marker in TNBC.^18^

IHC has become the gold standard method to assess protein expression in formalin-fixed, paraffin-embedded (FFPE) clinical samples. However, IHC typically relies on chromogenic detection that has a narrow linear dynamic range, thus limiting its ability to generate accurate quantitative results.^19^ Moreover, IHC scoring system is subjective and this can lead to reproducibility issues.^20,21^ Therefore, to improve the accuracy and reproducibility of HER family member quantification in tumours, we developed a time-resolved Förster resonance energy transfer (TR-FRET) method that allows the quantitative and objective measurements of protein expression levels. This method relies on the energy transfer between two fluorophores, a donor and an acceptor.^22^ When the donor (in this case, a lanthanide complex characterised by a long fluorescence lifetime) is excited by an energy source, it transfers its excitation energy to the acceptor only if the two fluorophores are in close proximity. The acceptor will then emit a specific long-lived fluorescence. In our assays, the analytes are detected using two antibodies that bind to two distinct epitopes within the analyte. One antibody is coupled to a donor fluorophore, while the second antibody is coupled to an acceptor fluorophore. When these antibodies bind to specific epitopes on the target protein, the distance between the donor and the acceptor is small enough to allow the energy transfer. The intensity of the acceptor fluorescence signal is proportional to the receptor number in the sample or standard, thereby allowing quantitative measurements. We previously described such assays for the quantification of HER1 and HER2 in tumour cryosections.^23,24^ As the use of these TR-FRET assays is limited by the need of fresh or freshly frozen tumour tissues, we now developed new TR-FRET assays to quantify HER1, HER2 and HER3 in FFPE tumour samples. In this study, we evaluated HER quantification by TR-FRET assays and assessed the prognostic role of HER1, HER2 and HER3 expression in TNBC.

## Methods

### Patients and tissue samples

Tumour samples were selected from the Montpellier Cancer Institute (ICM) biological resource centre. Pathological data including hormone-receptor status, HER2 status, histological type, grade and pTNM were recorded. Clinical data (e.g. age, type of treatment, occurrence and type of relapse) were obtained by review of the medical files. The selected population included patients with non-metastatic TNBC who underwent mastectomy or breast-conservative surgery with negative margins, without previous history of cancer. In total, 51 patients with TNBC treated between 2004 and 2008 were included in this retrospective study. These patients did not receive neoadjuvant treatment and were treated by postoperative radiotherapy or by radiotherapy with standard chemotherapy (anthracyclines and/or taxanes). All tumours were considered as ER- and PgR-negative and HER2-unamplified. ER and PgR status were assessed by IHC, using the mouse monoclonal antibodies 6F11 (against ER, 1:100 dilution, Leica, Wetzlar, Germany) and PgR636 (against PgR, 1:400 dilution, Dako, Glostrup, Denmark). ER and PgR negativity were defined as <10% of ER and PgR immunoreactivity, irrespective of the staining intensity. Of note, among the 51 ER-negative tumours, 5 included 1–10% of ER-stained tumour cells. HER2 status was determined by IHC using the anti-cErbB2 polyclonal antiserum (#A0485, 1:800 dilution, Dako). HER2 expression was scored according to the recommendations at the time of diagnosis.^25^ HER2 negativity was defined as a score of 0, 1+, or 2+, with normal gene copy number by fluorescent/chromogenic in situ hybridisation in tumours with a 2+ score.

### Tissue lysate preparation

For each FFPE tumour sample, a 3 µm–thick section was examined histologically after haematoxylin and eosin staining to assess the percentage of tumour cells. Adjacent 5 μm–thick sections were then macro-dissected to enrich in tumour cells (>50%) and used for lysate preparation. The number of sections was adjusted to the tumour area (ideally three sections for a tumour with an area of 100 mm² after macro-dissection). Sections were placed in a 1.5 ml microcentrifuge tube containing 1 ml of xylene substitute (#A5597, Sigma, Ile d’Abau, France). Paraffin was dissolved by incubation at room temperature (RT) for 5 min, and then removed by centrifugation. Pellets were washed to remove residual contaminants (absolute ethanol twice, then 95% ethanol), collected by centrifugation (12,000 RCF) and resuspended in 1X Tris/EDTA pH 9 (Target Retrieval Solution #S2367, Dako). Samples were then heated at 95 °C in a Thermomixer (Eppendorf, Hamburg, Germany) for 45 min, centrifuged to remove the Tris/EDTA buffer, and resuspended in 250 µl of Lysis Buffer (LB4 #64KL4FDF, Cisbio, Codolet, France). Finally, ice-cold samples were lysed by sonication (80 W for 15 s) using a Vibra-Cell™ sonicator (Sonics & Materials, Newtown, CT, USA). Lysates were clarified by centrifugation (10,000 RCF) at 4 °C for 5 min and then transferred to the wells of a microtiter plate.

### TR-FRET assays

The TR–FRET assays were carried out in white 384-well small volume high-base microtiter plates (Ref. 784075 Greiner Bio-One, Courtaboeuf, France) in duplicate using Eu^3+^ cryptate trisbipyridine (TBP, #62EUSPEA, Cisbio) as donor, and d2 as acceptor, conjugated to the specific antibodies. Antibodies were labelled as previously described.^24^ For HER1 quantification, the TR-FRET assay was performed using two monoclonal antibodies from the Total EGFR Cellular Assay Kit (#64NG1PEG, Cisbio). HER2 expression was quantified using Ab-15 (LabVision, Fremont, CA, USA) labelled with TBP, and Ab-8 (LabVision) labelled with d2. HER3 was quantified using the ErbB3 monoclonal antibody 2F12 (Thermo Scientific, Rockford, IL, USA) labelled with TBP, and the ErbB3 monoclonal antibody 2B5 (Thermo Scientific) labelled with d2. The final assay volume was 20 μl and comprised 16 μl of lysate and 4 µl of a solution containing the donor/acceptor antibodies diluted in HTRF cellular kinase detection buffer (Cisbio). Plates were incubated at RT for 20 h, and read using a Pherastar FS fluorometer (BMG LABTECH, Champigny-sur-Marne, France) with a classical HTRF protocol (excitation at 337 nm, donor and acceptor emission measured, respectively, at 620 nm and 665 nm, 60 μs delay, 400 μs integration). The TR-FRET signals (deltaF) were calculated as previously described.^26^

For each assay, a standard curve was prepared using human recombinant proteins to convert the TR-FRET signals into receptor number per µl of lysate. Recombinant HER1, HER2 and HER3 were purchased from Origene technologies (Catalog No.: TP710011; TP710032 and TP710089, respectively).

For normalisation to DNA concentration, 2 µl of the sample solution used for the TR-FRET assays was transferred into a new microplate well, and 18 µl of a solution containing 1X SYBR Safe DNA Gel Stain (Invitrogen, Carlsbad, CA, USA), 50 mM HEPES buffer and 0.1% BSA was added. After incubation at RT for 2 h, SYBR Green fluorescence intensity was measured by reading the fluorescence at 520 nm in Fluorescence Polarization mode upon 485 nm excitation using a Pherastar FS fluorimeter. Sample DNA concentrations were interpolated from a standard curve generated using serial dilutions in LB4 of a DNA standard of known concentration (50 µg/ml) prepared from human placental DNA (# D-7011, Sigma). By assuming 6.6 pg DNA per diploid cell, 1 ng of DNA was considered to correspond to 151 cells. This allowed converting the number of receptors per ng of DNA into a number of receptors per cell.

### Immunohistochemistry assays

IHC analysis was performed on 3 μm–thick FFPE tumour tissue sections. The HER1 and HER3 IHC assays were carried out on adjacent sections that are the mirror image of the ones used for the TR-FRET assays. For HER1 detection, slides were deparaffinised in xylene, hydrated in serial dilutions of alcohol, and then immersed in proteinase K solution (Dako) at RT for 10 min. For HER3 detection, section deparaffinisation, rehydration and antigen retrieval were performed simultaneously in an automated PT Link module (Dako) using EnVision FLEX Target Retrieval Solution, High pH (Dako), at 95 °C for 15 min. Endogenous peroxidase activity was blocked by incubation with the EnVision Flex Peroxidase Block (Dako) at RT for 5 min. Sections were then incubated with anti-HER1 (mouse monoclonal, clone 31G7, 1:50 dilution, Invitrogen) or anti-HER3 (mouse monoclonal, clone DAK-H3-IC, 1:50 dilution, Dako) antibodies, for 20 and 30 min, respectively. For HER3 detection, EnVision FLEX + Mouse LINKER (Dako) was used to amplify the primary antibody signal. After two rinses in EnVision FLEX wash buffer (Dako), sections were incubated with a horseradish peroxidase-labelled polymer coupled to secondary antibodies (Envision FLEX HRP, Dako) at RT for 20 min, followed by incubation with EnVision FLEX Substrate Working Solution containing 3,3’-Diaminobenzidine as chromogen (Dako) at RT for 10 min. Sections were counterstained with EnVision Flex Hematoxylin (Dako), rinsed with tap water for 5 min, dehydrated, and mounted with a coverslip. A negative control (a sample incubated with non-specific mouse IgG instead of the primary mouse monoclonal antibodies) was included in each IHC batch. Sections were read independently by two trained observers (Boissière-Michot F and Simony-Lafontaine J) blinded to the patients’ clinicopathological characteristics. HER1 and HER3 expression levels were scored using the H-Score method^27^ in which the membrane staining intensity (no staining = 0, weak staining = 1, moderate staining = 2 and intense staining = 3) is multiplied by the percentage of stained tumour cells (from 0 to 100%) to give a score ranging from 0 to 300. H-Scores were averaged between observers, except for conflicting results that were examined conjointly to reach consensus. HER2 expression levels were extracted from the patient medical files, and therefore, they were from IHC assays carried out using sections that were not the mirror image of the ones used for the TR-FRET assays.

### Statistical analysis

Continuous variables were described as medians and range, and categorical variables as frequencies and percentages. A non-parametric test for trend was performed to test the TR-FRET expression increase across the three ordered groups of expression determined by IHC (weak/negative, moderate, and high for HER1 and HER3; 0, 1+, and 2+ for HER2). Relapse-free survival (RFS) was defined as the time from the date of surgery to the date of last contact or recurrence (local, regional, or distant), and estimated using the Kaplan–Meier method. Univariate and multivariate analyses were performed using a Cox proportional hazard model. Hazard ratios (HR) were given with their 95% confidence interval (95% CI). All statistical analyses were performed with the STATA 13.0 software (StatCorp, College Station, TX).

## Results

### Patients and clinicopathological characteristics

The study cohort included 51 women with TNBC. Using 20 November 2014 as cut-off date, the median follow-up was 6.5 years (95% CI [5.3–7.8]). Among these patients, 19 (37.2%) had a recurrence that was loco-regional in nine and distant in ten patients. Most of these recurrences (84%) occurred during the first 42 months of follow-up, which is consistent with the previously reported relapse risk temporal distribution.^3^ The patients’ clinicopathological characteristics are detailed in Table 1.

**Table 1:** Patients’ characteristics.

Characteristics	Number of patients (%)
Age, years	
Median	62
Range	30–89
TNBC histological type	
Ductal	46 (90.2)
Other	5 (9.8)
Histologic grade (SBR)	
I	1 (2.0)
II	8 (15.7)
III	42 (82.3)
pT stage	
T1	14 (27.4)
T2	33 (64.7)
T3	3 (5.9)
T4	1 (2.0)
pN stage	
N–	25 (49.0)
N+	26 (51.0)

### TR-FRET assay performance

**Fig. 1:**
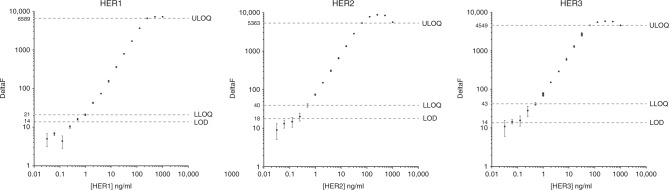
Calibration curve plots showing the limit of detection (LOD), lower limit of quantification (LLOQ), linear dynamic range, and upper limit of quantitation (ULOQ) of the HER1, HER2 and HER3 TR-FRET assays. For each assay, the LOD was calculated as the average signal of 20 replicate-negative samples plus three standard deviations. The lower limit of quantification (LLOQ) and the upper limit of quantification (ULOQ) were the lowest and highest standard point that can be measured with <15% coefficient of variance (CV).

The standard curves for TR-FRET-based quantification of HER expression showed a linear relationship between TR-FRET signal and HER1, HER2 and HER3 concentration for concentration ranges of 0.98–250 ng/ml (R² = 0.995), 0.24–62.5 ng/µl (R² = 0.997) and 0.24–62.5 ng/ml (R² = 0.988), respectively, and tended to saturate at higher levels (Fig. 1). The limit of detection (LOD) was in the range of 0.19–0.60 ng/ml, depending on the assay. The lower limit of quantification (LLOQ) values were 0.98 ng/ml (HER1), 0.48 ng/ml (HER2), and 0.48 ng/ml (HER3). For all assays, the upper limit of quantification (ULOQ) was equal to the concentration of the highest calibration standard within the linear dynamic range of concentrations (HER1: 250 ng/ml; HER2: 62.5 ng/ml; HER3: 62.5 ng/ml).

As the TR-FRET assays rely on antigen-antibody interactions, the specificity of each antibody pair needed to be validated. For that purpose, the ability of the antibody pairs to specifically detect HER1, HER2 or HER3 was tested by using as negative controls the other HER family members. These negative controls (1000 ng/ml of recombinant proteins) did not generate any TR-FRET signal above the LOD (data not shown).

### Precision of the TR-FRET assays

For each of the 51 TNBC samples, two independent technical replicates were prepared using consecutive serial sections from the same FFPE tumour block. Each lysate was independently used for HER1, HER2 and HER3 quantification by TR-FRET. Technical replicate data were normalised and the numbers of receptors per cell were then plotted to assess the precision of each assay. The values of duplicate samples were strongly correlated (HER1: R² = 0.986; HER2: R² = 0.993; HER3: R² = 0.877) (Supplementary Fig. 1). The median coefficient of variation values between technical replicates were 5.3% (HER1), 5.2% (HER2), and 8.2% (HER3). Taken together, these results demonstrate that the TR-FRET assays allow the reliable and precise quantification of HER1, HER2 and HER3 in FFPE tumour samples.

### HER1, HER2 and HER3 expression in TNBC

The IHC results are presented in Table 2 and in Supplementary Table 1. Briefly, among the 51 TNBC specimens, only seven (13.8%) displayed high HER1 expression. In most tumours, HER2 could not be detected (score = 0), and only 11 TNBC samples (21.5%) showed weak or moderate HER2 expression (score = 1+ or 2+). Moderate HER3 expression was found in nine tumours (17.6%), and none showed high HER3 expression. Representative examples of HER3 IHC staining are shown in Supplementary Fig. 2. Although only membrane staining was taken into account in the H-Score, HER3 membrane expression was significantly associated with cytoplasmic expression (*P* < 0.001). Indeed, 22 of the 30 HER3 membrane-positive tumours displayed concomitant cytoplasmic staining, whereas only 2 of the 21 HER3 membrane-negative TNBC samples showed HER3 cytoplasmic expression. HER3 nuclear expression was detected to various extent in 53% of TNBC specimens (27 of 51), without association with membrane or cytoplasmic HER3 expression (data not shown).

**Table 2:** HER1, HER2 and HER3 expression in TNBC by IHC.

	Number of patients (%)
HER1 Expression	
Weak (H-Score < 10)	22 (43.1%)
Moderate (10 ≤ H-Score < 150)	22 (43.1%)
High (H-Score ≥ 150)	7 (13.8%)
HER2 Expression	
0	40 (78.4%)
1+	9 (17.7%)
2+	2 (3.9%)
3+	0 (0%)
HER3 Expression	
Weak (H-Score < 10)	42 (82.4%)
Moderate (10 ≤ H-Score < 150)	9 (17.6%)
High (H-Score ≥ 150)	0 (0%)

**Fig. 2:**
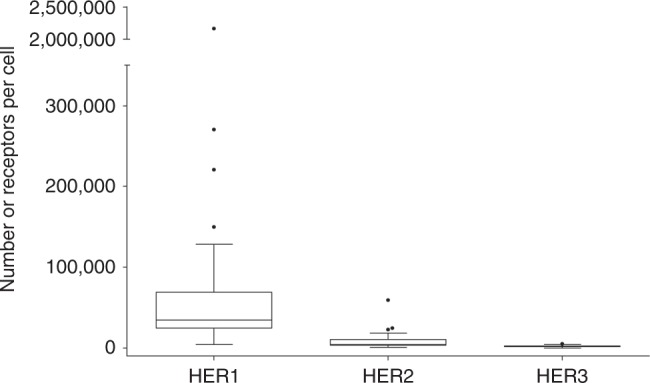
Quantitative measurement of HER1, HER2 and HER3 expression by TR-FRET in 51 TNBC samples.

The HER1, HER2 and HER3 expression levels measured by TR-FRET are shown in Fig. 2, and data are listed in Supplementary Table 1. The median number of receptors per cell were 34,540 (range 4452–2,159,308) for HER1, 4463 (range 945–59,302) for HER2, and 1532 (323–5348) for HER3. Low endogenous HER1, HER2 and HER3 levels (below 10,000 receptors per cell) were observed in 2 (3.9%), 37 (72.6%) and 51 (100.0%) tumours, respectively. Moderate expression (between 10,000 and 100,000 receptors per cell) of HER1 and HER2 was observed in 43 (84.3%) and 14 (27.4%) tumours, respectively. High HER1 expression (above 100,000 receptors per cell) was found in six (11.8%) tumours. Not surprisingly, in most tumours, HER1 expression levels were more elevated than those of HER2 and HER3 (96 and 100% of tumours, respectively).

Comparison of the TR-FRET and IHC results (Supplementary Fig. 3) showed a significant trend of increasing HER1 (*P* *<* 0.001) and HER2 (*P* *=* 0.007) receptor number by TR-FRET across the ordered IHC expression groups. For HER3, no significant trend was found between IHC and TR-FRET expression levels, because of its very low expression.

### Correlation with relapse-free survival

**Fig. 3:**
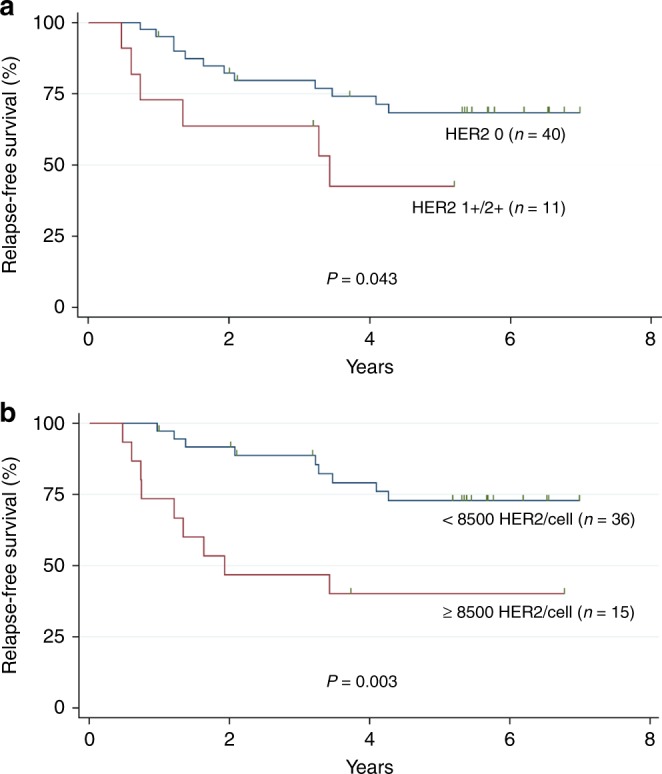
Kaplan–Meier curves for relapse-free survival of 51 patients with TNBC stratified according to HER2 expression by IHC (**a**) and by TR-FRET (**b**).

The estimated 36-month RFS rate was 76% (95% CI 62–86%). Univariate analysis showed that histological type, histologic grade and pathologic stage (pT) were not correlated with the RFS rate. Patients with node-positive (pN+) TNBC were more likely to relapse, although this trend did not reach significance (*P* = 0.056). Analysis of whether the HER1, HER2 or HER3 expression levels could be potential prognostic factors of relapse showed that moderate HER2 expression, assessed by IHC (*P* = 0.043) or TR-FRET (*P* = 0.003), was associated with significantly reduced RFS (Table 3 and Fig. 3). When variables with *p* < 0.1 were entered in multivariate analyses, only HER2 TR-FRET expression was an independent risk factor for tumour recurrence (Table 3).

**Table 3:** Univariate and multivariate analyses of prognostic factors related to relapse-free survival.

Prognostic marker	Events/patients (n)	Univariate analysis			Multivariate analysis		
		HR	95% CI	*p*^a^	HR	95% CI	*p*^*a*^
pN				0.056			
pN−	6/25	1					
pN+	13/26	2.47	0.94–6.51				
*HER2* expression (TR-FRET)				0.003			0.003
<8,500 receptors/cell	9/36	1			1		
≥8,500 receptors/cell	10/15	3.93	1.59–9.72		3.93	1.59–9.72	
*HER2* expression (IHC)				0.043			
0	12/40	1					
1+/2+	7/11	2.78	1.09–7.10				

HER2 expression levels measured by TR-FRET were dichotomised in the low and moderate expression groups according to the cut-off value derived from the ROC curve for predicting disease recurrence or metastases. The optimal cut-off value was 8,500 receptors per cell (sensitivity = 52.63%, specificity = 84.38%).

^a^Likelihood-ratio test.

## Discussion

Recently, we described a TR-FRET-based assay for the precise and objective quantitative analysis of HER family member expression in tumour cryosections.^23^ Here, we successfully applied this TR-FRET technology for the quantification of HER1, HER2 and HER3 in FFPE tumour samples.

The use of standard curves generated with recombinant HER1, HER2 and HER3 proteins allowed us to convert TR-FRET signals into number of receptors per µl of lysate. However, to accurately compare protein expression levels between patient samples using a quantitative method, measurements must be normalised relatively to the amount of starting material. Ideally, cell number should be assessed for each sample to derive the amount of proteins per cell. While this approach is feasible for cultured cells and blood samples, it is not applicable to tissue lysates. As DNA concentration is a robust surrogate of cell number in a wide range of cell types, including cancer cell lines,^28^ we developed a SYBR green-based quantification assay that measures the total DNA concentration in the same reaction mix used for the TR-FRET assay. Although most cancer cells are not diploid, we assumed that 1 ng of DNA was equivalent to 151 cells.^29^ For each assay, we then derived the number of receptors per cell from the number of receptors per ng of DNA. It is important to note that several factors could influence the determination of the receptor number per cell. First, chromosomal abnormalities are a hallmark of cancer cells, which means that tumour cells have variable DNA content. In addition, for tissue lysate preparation, samples undergo physical disruption in denaturing conditions that can damage HER protein epitopes. This implies that the measured receptor number is likely to represent an underestimation of the true receptor number. Finally, the extraction and solubilisation efficiency might be different between proteins and DNA. For these reasons, the calculated number of receptors per cell may differ from the true number of receptors per cell. Nevertheless, the TR-FRET signal conversion into number of receptors per cell is very convenient because it makes it easier to visualise the receptor expression level (low, moderate, or high) in a tumour. Moreover, the calculated number of receptors per cell was consistent from experiment to experiment, as indicated by the low coefficient of variation (CV < 10%) between technical replicates prepared from adjacent, mirror-image FFPE sections. This standardised relative quantification allows comparing the expression levels of different proteins, which is not possible with a semi-quantitative method, such as IHC.

We determined the performance of the new HER1, HER2 and HER3 TR-FRET assays for FFPE tumour samples by determining their LOD (from 0.19 to 0.60 ng/ml) and dynamic range (250-fold linear dynamic range). By comparison, commercially available ELISA kits, such as the Invitrogen human EGFR, ErbB2 and ErbB3 ELISA kits (Catalogue No.: KHR9061; EHERBB2 and EHERBB3, respectively), are more sensitive (<0.1 ng/ml), but with smaller dynamic range (less than 100-fold), and require much more biological material (sample volume is usually 100 µl vs 16 µl for our TR-FRET assays). Moreover, these ELISA kits are intended for serum and cell culture supernatants, and may not be suitable for HER1, HER2 or HER3 quantification in FFPE lysates.

When used to quantify HER1, HER2 and HER3 in 51 FFPE TNBC samples, our TR-FRET approach demonstrated a high reproducibility level (median CV of technical replicates ≤8%). This compares quite favourably with CV of ≈15% reported for other quantitative methods to measure protein expression in FFPE samples, such as the mTRAQ^30^ and VeraTag^TM^ proximity-based assays.^31^ We think that due to their precision, sensitivity and objectivity, our TR-FRET assays are an appropriate method for the quantification of HER family members.

The HER family plays a major role in the regulation of cell proliferation, differentiation, and survival (9), and aberrant HER signalling is frequent in breast cancer. To clarify the role of HER1, HER2 and HER3 in TNBC, we assessed the expression of these receptors in our 51 TNBC using IHC and compared the results with those obtained with the TR-FRET assays.

HER1 IHC overexpression has been reported in 13–76% of TNBC^32^ and is associated with worse disease-free survival.^10^ HER1 overexpression frequency depends strongly on the scoring methods and the antibodies used.^33^ In our study, we detected high HER1 expression only in 13.8% of TNBC samples by IHC with the anti-HER1 antibody clone 31G7 (Dako), which is widely used to assess HER1 expression in colorectal cancer, and the H-Score scoring method. We obtained similar results with the TR-FRET assay (11.8% of tumours with >100,000 HER1 receptors per cell). However, HER1 number per cell was higher than 10,000 HER1 in most TNBC samples, a value above the level that is usually considered as endogenous/physiological.^34,35^ These results nuance rather than contradict the belief that HER1 overexpression is common in TNBC.

Currently, HER2 status is generally determined by IHC. In our series of 51 TNBC, comparison of the TR-FRET and IHC results showed a significant trend of increasing HER2 receptor number by TR-FRET across the HER2 IHC ordered groups, from 0 to 2+. However, our HER2 TR-FRET assay could detect as few as 900 receptors per cell, whereas conventional IHC is typically 20 times less sensitive.^34^ HER2 expression values varied considerably among TNBC samples, with tumours displaying up to 60,000 receptors per cell. Therefore, in some TNBC, HER2 expression level is not negligible. Of note, the Ab-8 and Ab-15 antibodies used for HER2 TR-FRET quantification recognise epitopes localised in HER2 intracellular domain. This means that our HER2 TR-FRET assay also detects p95HER2, a truncated form of HER2 that lacks the extracellular domain.

HER3 overexpression has been associated with poor outcome in many cancer types.^36,37^ In our 51 TNBC samples, 82.4% displayed negative or weak HER3 expression by IHC, and all tested samples had less than 5500 receptors per cell by TR-FRET analysis. Our results indicate that most TNBC have very low HER3 expression.

Finally, analysis of HER1, HER2 and HER3 expression patterns relative to the patient outcome showed that HER2 level is a potential biomarker of relapse. Indeed, moderate HER2 expression by IHC (score of 1+/2+) and TR-FRET (≥8500 HER2 per cell) was significantly associated with reduced RFS in our cohort of 51 TNBC. These results are in accordance with previous findings showing that moderate HER2 expression (IHC score of 2+) correlates with relapse in a small series of 47 patients with TNBC.^17^ This suggests that HER2 could be an actionable target in TNBC with moderate HER2 expression levels. Two controversial studies published in 2008^13^ and 2010^14^ showed that some patients with HER2-negative breast tumours could benefit from HER2-targeting agents. Ithimakin et al. suggested that this unexpected finding might be explained by the cancer stem cell (CSC) hypothesis^38^ according to which CSCs are implicated in treatment resistance^39,40^ and tumour recurrence.^41^ Specifically, Ithimakin et al. demonstrated that, in some tumours with moderate HER2 levels, HER2 is selectively expressed in the CSC population. Therefore, trastuzumab and other HER2-targeting agents should be effective by targeting HER2-expressing CSCs. Indeed, trastuzumab can reduce the CSC populations of cell lines with moderate HER2 levels by specifically targeting HER2, and when administered early (adjuvant setting), it blocks the growth of tumour xenografts of these cell lines. Conversely, trastuzumab has no effect on cell lines and xenografts with low HER2 expression.^38^ However, the recent results of the NSABP B-47 trial undermine this theory by showing that adjuvant trastuzumab in patients with HER2 1+ or 2+ breast cancers by IHC does not improve disease- or relapse-free survival in this population.^16^ One possible explanation is that moderate HER2 expression is challenging to detect by IHC because of its semi-quantitative nature and its narrow dynamic range. In our study, multivariate analysis demonstrated that HER2 protein expression determined by TR-FRET is a stronger predictor of relapse than the HER2 IHC score. However, these results have some limitations due to the small patient population and the retrospective nature of our study. Prospective studies with larger cohorts of patients are required to confirm the relevance of our results.

Currently, IHC is the gold standard method to assess protein expression in patient tissue samples; however, its reproducibility is still an issue, although many improvements have been made in the past several years.^21^ Alternative methods exist for the objective quantification of HER proteins in FFPE samples. First, IHC can be enhanced by using fluorescence microscopy and advanced image analysis algorithms. This approach allows the continuous and quantitative measure of protein expression, but the inherent autofluorescence of FFPE sections may be a source of trouble.^42^ Recently, Targeted Mass Spectrometry has been used to assess HER2 expression in FFPE tissue samples.^43^ This approach is more quantitative than IHC and does not depend on the availability of specific antibodies. However, the detection of low and moderate levels of proteins remains challenging. Alternatively, Monogram Biosciences proposes the VeraTag™ proximity-based assays^44,45^ to quantify HER1, HER2 and HER3 in FFPE samples. The VeraTag™ technology uses a dual antibody format, whereby a fluorescent tag on one anti-HER antibody is released when in close proximity to a second specific antibody conjugated with molecular scissors that are activated upon illumination. The VeraTag™ assays are very sensitive and can detect down to 2500 receptors per cell.^46^ However, access to this technology for research purposes is restricted because samples must be shipped to the Monogram CAP/CLIA certified laboratory in California for the analysis. We believe that our TR-FRET approach is a suitable alternative to these methods, because it overcomes many of their limitations. By using long-lived fluorophores, a delay can be introduced between the excitation pulse and the signal measurement window, thus allowing the elimination of short-lived background autofluorescence from FFPE material. Our assays are quantitative, with high sensitivity, and could be easily performed in any laboratory equipped with a TR-FRET instrument, without much training required. Moreover, the TR-FRET technology could be extended to the quantification of other proteins of interest, such as p95HER2, the relevance of which as a prognostic marker has recently been reported in patients with HER2-positive breast cancer treated with trastuzumab.^47,48^

In summary, the present study demonstrates that our TR-FRET assays can reproducibly quantify the expression of HER family members in FFPE samples with high sensitivity. Moreover, it shows that quantification of HER2 expression by TR-FRET may be useful to predict tumour recurrence in TNBC, although additional studies in a larger population are required to confirm our findings.

## Supplementary information

Supplemental Material

## Data Availability

The datasets generated and/or analysed during the current study are available from the corresponding author on reasonable request.
