# Supplementary material for: Quantification of HER1, HER2 and HER3 by time-resolved Förster resonance energy transfer in FFPE triple-negative breast cancer samples
Source: Br J Cancer. 2019 Dec 3;122(3):397–404. doi: 10.1038/s41416-019-0670-8 (PMC7000684; doi:10.1038/s41416-019-0670-8)
Supplement: Supplementary file 1 — Supplemental Material [file 41416_2019_670_MOESM1_ESM.pdf]

Supplementary Fig. 1. Scatter plots showing the distribution of HER1 (a), HER2 (b) and HER3 (c) values (number of receptors per cell) between technical replicates of FFPE TNBC sample lysates. For HER1, one tumour that expresses more than 2 million receptors per cell (CV = 11.1%) was excluded from the linear regression analysis.

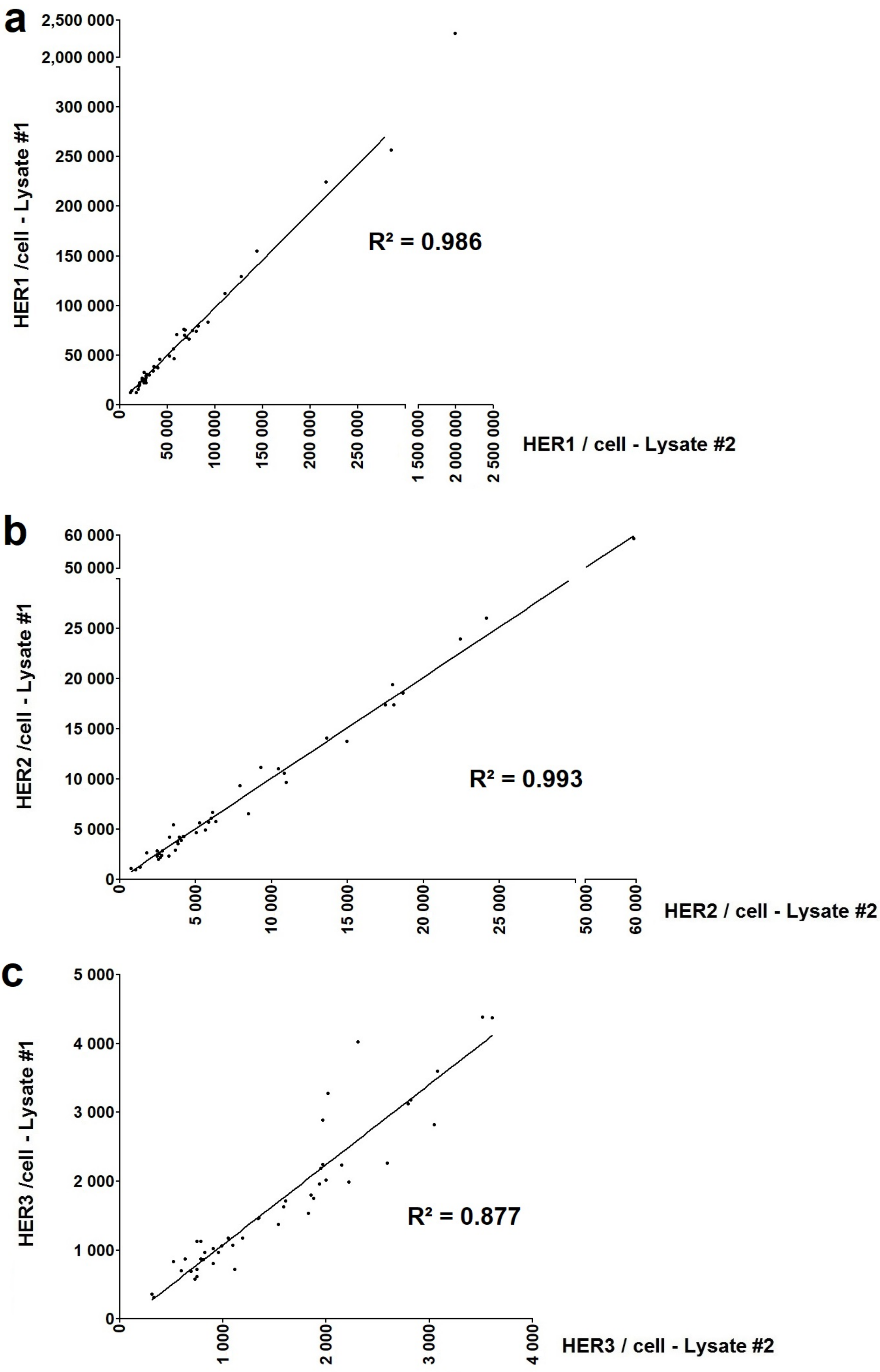

Supplementary Fig. 2. Representative examples of immunohistochemistry analysis of HER3 expression in FFPE breast cancer tissue sections *(x400)*. **High HER3 expression was observed only in HER2-amplified control samples, such as the one shown to the right panel**.

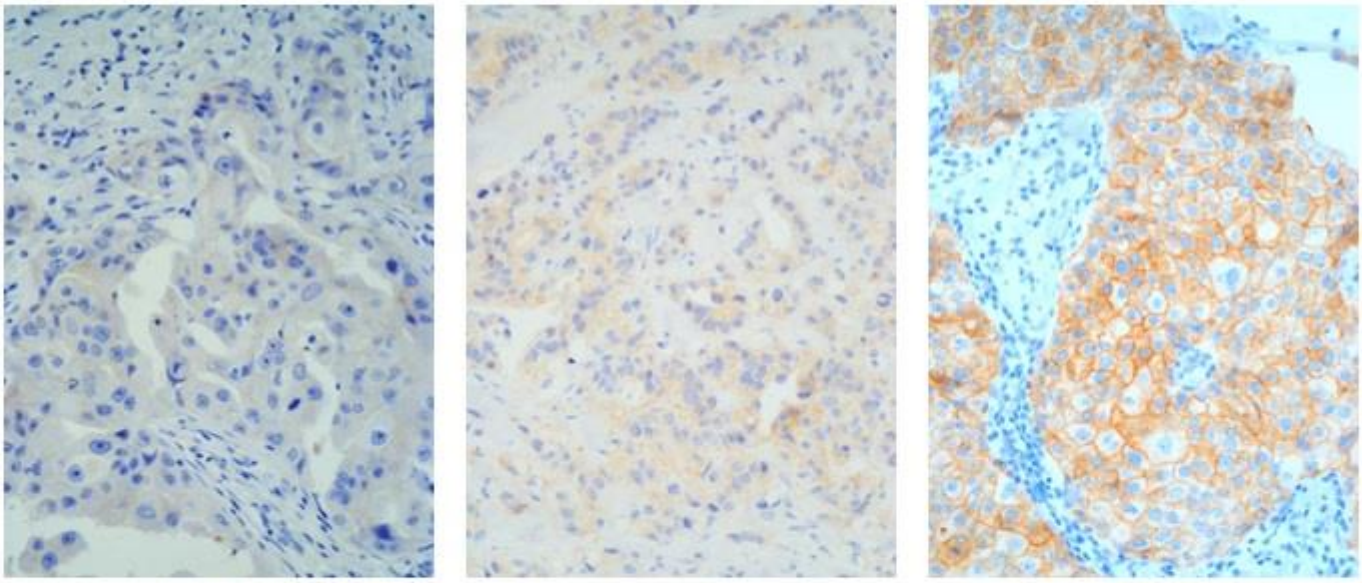

Negative/weak HER3 expression | Moderate HER3 expression | High HER3 expression

Supplementary Fig. 3. Comparison of HER1 (a), HER2 (b) and HER3 (c) expression assessed by TR-FRET assays and IHC.

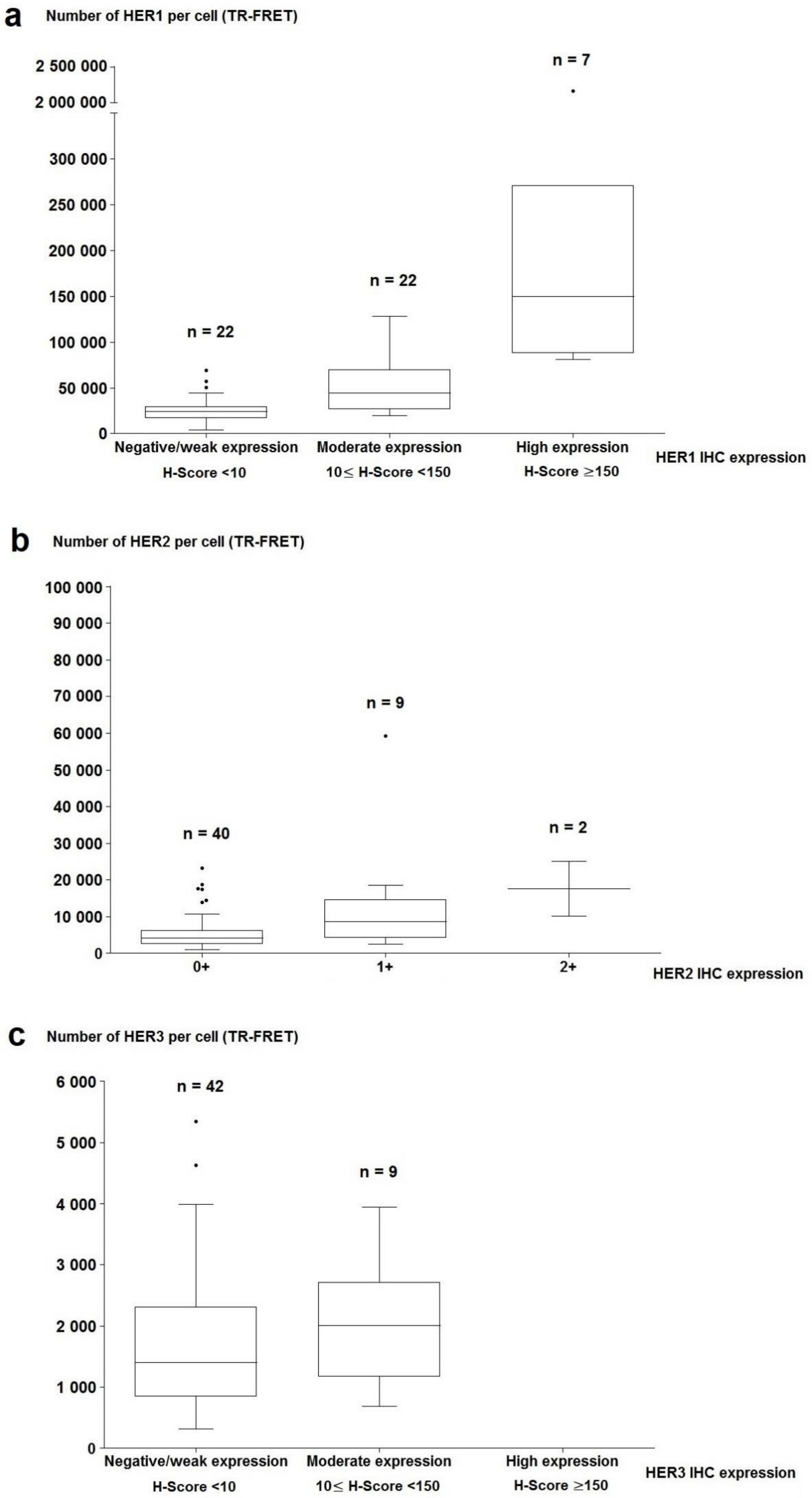

Supplementary Table 1. Detailed biological and clinical data

Sample	HER1 IHC expression (H-Score)	HER2 IHC expression	HER3 IHC expression (H-Score)	HER1 TR-FRET expression (receptors / cell)	HER2 TR-FRET expression (receptors / cell)	HER3 TR-FRET expression (receptors / cell)	Patient age (years)	TNBC histological type	Histologic grade (SBR)	pT stage	pN stage	Adjuvant treatment	Relapse
Sample 1	4,5	0	0	23 975	3 714	1 824	40	Other	III	T1	N+	Anthracyclines + Radiotherapy	Yes
Sample 2	170	0	40	220 545	4 514	935	62	Ductal	II	T2	N+	Anthracyclines + Radiotherapy	Yes
Sample 3	0	0	1.5	24 116	17 477	2 935	62	Ductal	II	T2	N+	Anthracyclines + Taxanes + Radiotherapy	Yes
Sample 4	5	0	0	28 675	4 232	962	44	Ductal	III	T1	N+	Anthracyclines + Radiotherapy	No
Sample 5	5	0	0	27 560	2 447	756	42	Ductal	III	T1	N-	Anthracyclines + Radiotherapy	No
Sample 6	0	0	35	11 809	18 691	1 410	83	Ductal	III	T2	N+	Radiotherapy	Yes
Sample 7	0	1+	0.25	4 452	59 302	825	63	Ductal	II	T2	N+	Anthracyclines + Taxanes + Radiotherapy	Yes
Sample 8	0,75	0	2.5	24 737	4 274	1 609	48	Ductal	III	T2	N+	Anthracyclines + Taxanes + Radiotherapy	No
Sample 9	130	0	0.25	128 261	3 797	1 086	86	Ductal	III	T4	N-	Radiotherapy	Yes
Sample 10	15,25	1+	0	29 076	8 625	959	77	Ductal	III	T2	N-	Radiotherapy	Yes
Sample 11	20	0	0.125	25 017	5 454	840	46	Ductal	III	T2	N-	Anthracyclines + Radiotherapy	No
Sample 12	0	0	1	13 651	10 719	2 104	78	Ductal	II	T2	N+	Radiotherapy	Yes
Sample 13	200	0	0	87 977	2 305	3 166	64	Ductal	III	T1	N-	Radiotherapy	Yes
Sample 14	110	1+	0	77 138	10 337	654	66	Ductal	III	T2	N-	Taxanes + Radiotherapy	No
Sample 15	20	0	0	72 059	5 785	855	73	Ductal	III	T2	N+	Anthracyclines + Taxanes + Radiotherapy	No
Sample 16	75	0	1.25	34 621	2 683	737	77	Ductal	III	T3	N+	Radiotherapy	Yes
Sample 17	17,5	1+	112.5	34 540	2 582	2 006	53	Ductal	III	T2	N+	Anthracyclines + Taxanes + Radiotherapy	No
Sample 18	60	1+	5	68 982	6 077	1 028	68	Ductal	III	T2	N-	Anthracyclines + Radiotherapy	No
Sample 19	40	0	0.05	50 838	3 301	686	47	Ductal	III	T2	N-	Anthracyclines + Taxanes + Radiotherapy	No
Sample 20	30	0	0	37 300	4 871	1 184	61	Ductal	III	T2	N-	Anthracyclines + Radiotherapy	No
Sample 21	30	0	5	71 640	13 841	1 682	64	Ductal	III	T2	N+	Radiotherapy	No
Sample 22	50	0	0	25 946	2 840	1 115	37	Ductal	III	T1	N-	Anthracyclines + Radiotherapy	No
Sample 23	160	0	0.125	81 163	4 463	2 193	62	Ductal	I	T2	N+	Anthracyclines + Taxanes + Radiotherapy	No
Sample 24	5	1+	20	24 930	2 933	1 950	75	Ductal	III	T1	N-	Radiotherapy	Yes
Sample 25	0,5	0	5.5	30 637	4 094	962	72	Ductal	III	T2	N-	Radiotherapy	No
Sample 26	65	0	0	26 951	14 380	2 638	47	Other	II	T1	N-	Anthracyclines + Radiotherapy	No
Sample 27	300	0	0	2 159 308	2 808	917	83	Ductal	III	T2	N-	Radiotherapy	No
Sample 28	0	0	0	7 238	1 294	1 456	79	Ductal	III	T2	N+	Radiotherapy	Yes
Sample 29	60	0	10	51 685	2 597	2 427	49	Ductal	III	T3	N+	Anthracyclines + Taxanes + Radiotherapy	No
Sample 30	10	1+	75	24 040	10 732	2 999	89	Ductal	III	T2	N+	Radiotherapy	Yes

Sample 31	4,5	0	0	20 362	3 759	896	44	Ductal	III	T2	N+	Anthracyclines + Taxanes + Radiotherapy	Yes
Sample 32	180	1+	10	111 197	18 618	2 066	70	Ductal	III	T2	N+	Anthracyclines + Taxanes + Radiotherapy	Yes
Sample 33	0	0	0.5	50 507	10 224	1 904	51	Other	III	T2	N+	Anthracyclines + Taxanes + Radiotherapy	No
Sample 34	3	0	0	14 962	7 503	1 403	63	Ductal	III	T1	N-	Anthracyclines + Taxanes + Radiotherapy	No
Sample 35	1,5	0	2.5	21 691	17 717	3 338	37	Ductal	II	T2	N-	Anthracyclines + Radiotherapy	No
Sample 36	1	2+	0	57 394	25 094	4 630	85	Other	II	T2	N-	Radiotherapy	Yes
Sample 37	30	0	0	38 584	6 052	1 660	47	Ductal	III	T1	N+	Anthracyclines + Taxanes + Radiotherapy	No
Sample 38	95	0	1	56 423	1 076	1 815	68	Ductal	III	T3	N+	Anthracyclines + Taxanes + Radiotherapy	No
Sample 39	30	0	0	75 658	945	675	38	Ductal	III	T2	N-	Anthracyclines + Taxanes + Radiotherapy	Yes
Sample 40	210	0	0.5	149 724	23 182	4 354	58	Ductal	III	T2	N+	Anthracyclines + Taxanes + Radiotherapy	Yes
Sample 41	4	2+	0	28 728	10 241	650	78	Ductal	III	T2	N+	Radiotherapy	Yes
Sample 42	70	0	0	24 868	2 207	2 105	88	Ductal	II	T1	N-	Radiotherapy	No
Sample 43	7,5	0	0	69 096	2 568	2 649	61	Other	III	T2	N-	Anthracyclines + Taxanes + Radiotherapy	No
Sample 44	1,5	0	7	23 893	6 386	3 992	69	Ductal	III	T2	N-	Anthracyclines + Taxanes + Radiotherapy	No
Sample 45	180	0	87.5	270 725	4 000	3 948	82	Ductal	III	T1	N-	Radiotherapy	No
Sample 46	0	0	2.5	17 648	4 771	5 348	55	Ductal	III	T1	N+	Anthracyclines + Taxanes + Radiotherapy	No
Sample 47	110	0	0	65 271	4 124	2 957	36	Ductal	III	T1	N-	Anthracyclines + Taxanes + Radiotherapy	No
Sample 48	10	0	0.5	19 600	1 029	336	74	Ductal	III	T2	N+	Anthracyclines + Radiotherapy	No
Sample 49	1	0	0.75	44 046	1 282	323	81	Ductal	III	T2	N-	Radiotherapy	No
Sample 50	5,5	0	50	29 439	2 419	694	31	Ductal	III	T2	N+	Anthracyclines + Taxanes + Radiotherapy	No
Sample 51	110	1+	0.5	69 428	5 305	2 427	47	Ductal	III	T1	N-	Anthracyclines + Taxanes + Radiotherapy	No
